# 1026. Case Study. Collaborative Oral Care Protocol in a Burn Patient with Complex Perioral Contracture

**DOI:** 10.1093/jbcr/irag033.525

**Published:** 2026-04-06

**Authors:** Michelle N Dwertman, Victoria Allen, Catherine Freeman

**Affiliations:** University of Cincinnati Medical Center Burn Center, Cincinnati, OH; University of Cincinnati Medical Center, Cincinnati, OH; University of Cincinnati Medical Center, Cincinnati, OH

## Abstract

**Patient Presentation (age range, injury details, relevant history):**

Oral health complications following facial burns are an underrecognized barrier to recovery. Perioral contractures, microstomia, mucosal sensitivity, and limited dental access can contribute to infection risk, impaired nutrition, and delayed rehabilitation. This case highlights the use of a collaborative oral care protocol, developed between burn therapy and a university Dental Center, to address these challenges.

**Case Presentation:**

Pt is a 33-year-old. Male with 46% TBSA to the face, neck, chest, both arms, hands, bilateral thighs and back. Pt was in a demolition derby when his vehicle collided with another, and his car caught fire. Escharotomies were completed on both arms and was intubated on ECMO then stabilized. The patient was then extubated, received a skin substitute and mesh grafting to chest, bilateral thighs and back and sheet autograft to both hands and neck. His face healed in without grafting.

**Clinical Challenges:**

The partial thickness burns to the lower face and perioral region, resulted in microstomia, and limited maxillary mobility. Standard oral hygiene was compromised due to tooth and oral pain, restricted mouth opening, and inability to tolerate conventional toothbrushes. Delays in dental access further elevated his risk for increased tooth decay and periodontal infection.

**Management Approach:**

Under burn surgeon approval, a modified oral care protocol was initiated in collaboration with a university dental program. Daily practices included the use of a pediatric soft-bristled toothbrush for dental hygiene combined with intraoral stretching, desensitizing toothpaste, alcohol-free fluoride mouthwash, and adaptive flossing devices. The Burn therapists provided targeted exercises to improve oral aperture. To bridge the gap until formal dental treatment could begin, topical fluoride varnish was applied by trained burn staff every three months, following a standardized technique to optimize enamel protection.

**Outcomes:**

After eight weeks, the patient demonstrated improved mouth opening (+12 mm interincisal distance), reported reduced oral sensitivity, and showed no evidence of new carious lesions or gingival infection. The integrated protocol supported functional oral maxillary recovery and prevented secondary complications.

**Lessons Learned:**

This case illustrates the value of an interdisciplinary approach to post-burn oral and maxillary care. Incorporating adapted hygiene tools, therapeutic exercises, and in-clinic fluoride application can mitigate infection risk and preserve oral health in patients with facial maxillary burns, especially when immediate dental services are unavailable.

**Applicability to Practice:**

Burn team members can play a integral role in bridging the gap between burn care, oral hygiene by incorporating a collaborative approach to oral care education, adaptive hygiene tools, and interim fluoride applications to meet patient needs throughout post-burn recovery.

